# A Case of Peritoneal Tuberculosis Mimicking Ovarian Cancer in a Young Female

**DOI:** 10.1155/2022/4687139

**Published:** 2022-09-28

**Authors:** Oriel Nissim, F. Richard Ervin, Susan E. Dorman, Deeksha Jandhyala

**Affiliations:** ^1^Medical University of South Carolina, Charleston, South Carolina, USA; ^2^South Carolina Department of Health and Environmental Control, South Carolina, USA

## Abstract

**Background:**

Tuberculosis causes significant morbidity and mortality globally. Peritoneal tuberculosis can have a similar presentation to ovarian cancer.

**Case:**

We present a case of a 42-year-old female referred to gynecology oncology with imaging findings of enlarged right ovary, omental caking, and elevated CA-125 (1289 U/mL). A diagnostic laparoscopy revealed diffuse studding of intraperitoneal surfaces. Histopathological examination of omental and abdominal wall biopsies showed granulomas, but stains and cultures for mycobacteria were negative. Antimicrobial treatment for tuberculosis was initiated. Within eight weeks, there was clear clinical and radiographic improvement, consistent with a diagnosis of peritoneal tuberculosis.

**Conclusion:**

This case highlights the importance of including peritoneal tuberculosis in the differential diagnosis when evaluating for ovarian cancer in women with epidemiologic risk factors for tuberculosis.

## 1. Introduction

Tuberculosis is a leading cause of morbidity and mortality on a global level, with approximately 10 million incident cases and 1.3 million deaths annually [1]. Tuberculosis is relatively uncommon in the United States (2.7 case rate/100,000 population in 2019) and typically presents as a lung infection, although it can affect any organ system [2].

Here, we describe peritoneal tuberculosis in a young female from Brazil who presented with elevated CA-125 and radiological findings concerning for ovarian malignancy.

## 2. Case Presentation

A 42-year-old G0 female was referred to the gynecology oncology outpatient clinic for evaluation of symptoms and imaging findings concerning for ovarian cancer. She reported six months of abdominal discomfort and distension, night sweats, fever, and weight loss. Her previous gynecologic history included a ruptured ovarian cyst five years prior, but medical records were not available for review. She had no chronic medical conditions and did not take any medications. She had been born and lived in Brazil until moving to the U.S. three years prior to the current presentation. In Brazil, she had worked in a public health clinic that provided tuberculosis care; she had never been evaluated for *M. tuberculos*is infection or tuberculosis. On physical examination, she was generally well-appearing. The abdomen was distended but soft and nontender to palpation. External genitalia, vagina, and cervix had normal appearance; the uterus and cervix were deviated to the right on exam, and rectovaginal examination was normal. There was no thrush, peripheral lymphadenopathy, or lower extremity edema; heart and lung exams were normal.

**Figure 1:**
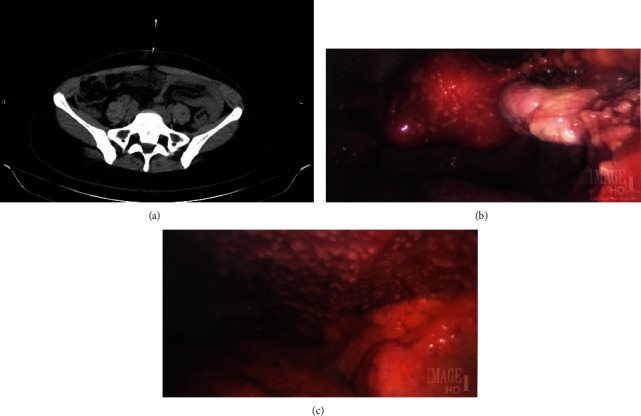
(a) Computed tomography (CT) imaging of the pelvis at the time of presentation and needle biopsy of omental caking. (b) Diagnostic laparoscopy showing innumerable tan, 1-2 mm nodules on the surface of the right ovary and uterus. (c) Diagnostic laparoscopy showing innumerable tan, 1-2 mm nodules on the anterior abdominal wall.

CT with contrast of the abdomen and pelvis showed a moderate volume of free fluid throughout the abdomen with diffuse nodularity throughout the omentum, enlarged right retroperitoneal lymph nodes measuring 1.4 and 1.2 cm in the short axis diameter, and a bilobed right ovarian cyst measuring 6.5 cm at maximum diameter (Figure 1(a)). A transvaginal ultrasound showed a hypoechoic lesion with low-level internal reticular echoes within the right ovary, consistent with a hemorrhagic cyst (which resolved based on follow-up ultrasound examination); an 0.8 cm hypoechoic structure in the endometrium; and cystic structures throughout the myometrium. A test for antibodies to HIV-1 and HIV-2 was nonreactive. Complete blood count and metabolic panel were normal. Blood CA-125 level was 1289 U/mL (normal ≤ 38.1 U/mL).

Paracentesis was performed and showed rare benign mesothelial cells. Endometrial biopsy showed secretory endometrium without evidence of malignant cells. A CT-guided omental biopsy showed granulomas without evidence of neoplasm; mycobacterial and fungal stains and cultures were negative. Diagnostic laparoscopy was performed. Upon inspection, there was diffuse studding of intraperitoneal surfaces with 1-2 mm tan nodules, and the omentum was adherent to the anterior abdominal wall (Figures 1(b) and 1(c)). There was an enlarged uterus with multiple fibroids, and the ovaries were without masses. Pathology of the omental and abdominal wall biopsies showed benign fibroadipose tissue with necrotizing and nonnecrotizing granulomas but without acid fast bacilli seen on modified Ziehl-Neelsen staining; mycobacterial cultures using the Mycobacteria Growth Indicator Tube (MGIT) liquid culture system and Lowenstein-Jensen solid media were negative, and nucleic acid amplification testing was negative for *Mycobacterium tuberculosis*.

Due to unknown cause of intraperitoneal granulomas, she was referred to an infectious disease physician and to the local health department. CT of the chest showed a 4 mm left lower lobe nodule and biapical mild pleural parenchymal scarring. A tuberculin skin test showed 10 mm induration, and a blood QuantiFERON-TB Gold Plus interferon-gamma release assay (Qiagen) was positive. Due to concern for peritoneal tuberculosis, treatment was initiated with isoniazid, rifampin, pyrazinamide, and ethambutol. Within four weeks of treatment, the CA-125 had decreased to 74 U/mL, and she had substantial clinical improvement including resolution of fevers, reduction in abdominal discomfort, increased appetite, and weight gain. At eight weeks after tuberculosis treatment initiation, her abdominal pain was entirely resolved, weight had increased from 56.6 kg at presentation to 62.1 kg, and CT abdomen and pelvis showed resolution of omental caking, ascites, and lymphadenopathy. A clinical diagnosis of tuberculosis peritonitis was made, and standard tuberculosis treatment was administered for a total of 6 months.

## 3. Discussion

Tuberculosis is caused by the bacterium *Mycobacterium tuberculosis* and typically manifests as a pulmonary infection. However, tuberculosis can affect any organ system, and from 10 to 42% of all tuberculosis patients have disease outside the respiratory tract (so-called “extrapulmonary” tuberculosis) without or with pulmonary tuberculosis [3]. Peritoneal tuberculosis is uncommon, with most cases reported in countries with a high overall prevalence of tuberculosis including India and China [4, 5]. Tuberculous peritonitis is thought to arise most commonly from activation of latent foci of *M. tuberculosis* infection in the peritoneum and less commonly from direct spread from genitourinary sites or hematogenous spread of bacteria from active pulmonary lesions [6]. A high index of suspicion is required since tuberculous peritonitis can present insidiously and with a variety of systemic and organ-specific signs and symptoms that can mimic other conditions.

Our patient's presentation with radiographic imaging suggestive of ovarian carcinomatosis and elevated CA-125 levels is typical. CA-125, a mucin-type glycoprotein that is encoded by the MUC16 gene and associated with the cell membrane, is an imperfect biomarker for ovarian cancer due to shortcomings in sensitivity and specificity [7]. Elevated CA-125 levels can be observed in physiological (e.g., menstruation and pregnancy) and pathological conditions, particularly inflammatory diseases of the peritoneum including tuberculosis, endometriosis, pelvic inflammatory disease, adenomyosis, and peritoneal sarcoid [8].

Laparoscopy has emerged as an important tool for diagnosis of peritoneal tuberculosis as it permits direct visualization as well as biopsy of affected tissues [9, 10]. The characteristic laparoscopic visual finding is diffuse studding of the visceral and parietal peritoneum with tan nodules as in this patient; turbid ascites and adhesions can also be present. Although laparoscopy is important in diagnosing peritoneal tuberculosis, the visual view can be deceiving even to experienced clinicians, since laparoscopic features of tuberculosis can mimic those of disseminated abdominal malignancies such as can occur with ovarian cancer [11].

In this patient, an epidemiologic clue was her prior residence in Brazil and work there in a public health clinic that provided tuberculosis care. In addition, she had evidence of prior *M. tuberculosis* infection on chest CT scan, but no evidence of ongoing active pulmonary tuberculosis. The positive delayed-type hypersensitivity response to intradermal inoculation of purified protein derivative (tuberculin skin test) and elaboration of interferon-gamma by peripheral blood cells stimulated with *M. tuberculosis*-specific antigens (interferon-gamma release assay) also provided evidence of prior *M. tuberculosis* infection. Granulomas on histopathologic examination were strongly supportive of the diagnosis of tuberculosis peritonitis. While mycobacterial culture and nucleic acid amplification testing of affected lesions should be performed in order to obtain a definitive microbiological diagnosis and antimicrobial susceptibility information, the sensitivity of these assays is imperfect due to low bacterial burden and/or sampling error; histopathology remains the diagnostic gold standard [12, 13]. This patient had clinical, radiographic, and biomarker response to antituberculous therapy.

In sum, this report describes a woman with tuberculous peritonitis, an entity seen uncommonly in areas of low TB prevalence such as the United States. This case exemplifies the multidisciplinary effort needed to identify tuberculous peritonitis, an often-elusive diagnosis, and highlights the critical role that gynecologists play when this disease occurs in women.

## Data Availability

Data supporting the report's conclusions are contained within the report. Additional nonrelevant patient data are protected under patient privacy regulations and policies.

## Additional Points

*Teaching Points*. (i) Peritoneal tuberculosis typically presents with signs, symptoms, and imaging findings that can mimic those of ovarian cancer. (ii) Peritoneal tuberculosis can cause a markedly elevated CA-125. (iii) Peritoneal tuberculosis should be in the differential diagnosis when evaluating for ovarian cancer in women with epidemiological risk factors for tuberculosis infection. Biopsies should be sent for histopathology as well as mycobacterial cultures.
